# Impact of peer feedback on the performance of lecturers in emergency medicine: a prospective observational study

**DOI:** 10.1186/s13049-014-0071-1

**Published:** 2014-12-04

**Authors:** Miriam Ruesseler, Faidra Kalozoumi-Paizi, Anna Schill, Matthias Knobe, Christian Byhahn, Michael P Müller, Ingo Marzi, Felix Walcher

**Affiliations:** Department of Trauma Surgery, University Hospital, Johann Wolfgang Goethe-University, Theodor Stern Kai 7, 60590 Frankfurt, Germany; Department of Trauma Surgery, University Hospital RWTH Aachen, Pauwelstr. 30, 52047 Aachen, Germany; Department of Anesthesiology, Pain Therapy and Intensive Care Medicine, European Medical School Oldenburg-Groningen, Evangelisches Krankenhaus, Steinweg 13-17, 26122 Oldenburg, Germany; Department of Anesthesiology and Critical Care Medicine, Carl Gustav Carus University Hospital, Fetscherstr. 74, 01307 Dresden, Germany; Department of Trauma Surgery, Otto-von-Guericke-University, Leipziger Street 44, 39120 Magdeburg, Germany

**Keywords:** Peer feedback, Lecture, Faculty development, Emergency medicine, Undergraduate education

## Abstract

**Background:**

Although it is often criticised, the lecture remains a fundamental part of medical training because it is an economical and efficient method for teaching both factual and experimental knowledge. However, if administered incorrectly, it can be boring and useless.

Feedback from peers is increasingly recognized as an effective method of encouraging self-reflection and continuing professional development. The aim of this observational study is to analyse the impact of written peer feedback on the performance of lecturers in an emergency medicine lecture series for undergraduate students.

**Methods:**

In this prospective study, 13 lecturers in 15 lectures on emergency medicine for undergraduate medical students were videotaped and analysed by trained peer reviewers using a 21-item assessment instrument. The lecturers received their written feedback prior to the beginning of the next years’ lecture series and were assessed in the same way.

**Results:**

In this study, we demonstrated a significant improvement in the lecturers’ scores in the categories ‘content and organisation’ and ‘visualisation’ in response to written feedback. The highest and most significant improvements after written peer feedback were detected in the items ‘provides a brief outline’, ‘provides a conclusion for the talk’ and ‘clearly states goal of the talk’.

**Conclusion:**

This study demonstrates the significant impact of a single standardized written peer feedback on a lecturer’s performance.

## Background

The lecture represents an economical and efficient method of conveying both factual and experiential knowledge to a large group of students and thus remains a fundamental part of the learning experiences of students during their medical education [1-5]. In their AMEE Medical Education Guide No. 22, Brown and Monogue conclude based on a review of the research on lecturing over the past 70 years that lectures are at least as effective for presenting and explaining conceptual and systematic knowledge and fostering enthusiasm and motivation for learning as other teaching methods [1]. Lectures offer a real-time, human presence with spoken communication, which for most people, is easy to learn from [2].

However, lectures, like all teaching methods, have their limitations and if administered incorrectly, can be boring and or even worse, useless. Although this didactic format is widely used and familiar to audiences, the skills required to prepare and to deliver an effective and structured lecture are mostly passed along through experiential learning and only seldom acquired by specific instruction in teaching techniques [1]. However, many studies exist that describe strategies for improving lecture presentation, ultimately increasing student learning [1,4,6,7].

Reflection on practice is the cornerstone and most powerful source of continuing professional development in all teaching environments, but reflection on practice and change requires insight, effort, and a willingness to change [1,8]. Although an educator’s teaching is mostly assessed by students, there is growing consent that effective assessment of teaching must emerge from multiple sources, especially peers, to provide essential data [9-13]. Feedback from peers and professional staff (faculty) developers is increasingly recognized as a valuable adjunct to surveys of student opinion. Such feedback can provide insights not possible based on student opinion alone. Effective peer assessment of teaching should be criteria-based, emphasize teaching excellence and use instruments that produce highly reliable measures [9,14].

The aim of this study is to evaluate the effect of a standardized written peer assessment on the quality of a lecture series in emergency medicine for undergraduate medical students. Several studies have reported the development of instruments for peer assessment and assessed their feasibility and reliability in pilot runs [7,12,14,15]; however, only McLeod et al. have described in a recent qualitative study the perceptions, benefits and shortcomings of peer assessment of reviewers and individuals reviewed [16]. However, this is the first study we are aware of with the intention to analyse the impact of peer assessment in a lecture series on the lecture itself.

## Methods

### Study design and ethics statement

This study has a prospective design in order to analyse the impact of written peer assessment based on a quantitative questionnaire about the lecturers’ performances in a lecture series in emergency medicine.

As stated by the Ethics board of the medical faculty of J.W. Goethe University Hospital, Frankfurt, Germany, ethical approval was not required for this study. The research of educational methods is required in the regulations on the licence to practice medicine in Germany and is supported by the medical faculty.

### Participants

The study participants were physicians from different disciplines who as part of their function as a medical teacher participate as lecturers in the lecture series on emergency medicine for undergraduate medical students at Johann Wolfgang Goethe University, Frankfurt/Main, Germany.

Data were obtained from all lecturers regarding age, years of lecturing experience, and training in medical education (e.g. Instructor training). Prior to the beginning of this study, all of the participants provided written informed consent to participate in this study and to be videotaped during their lectures.

### Study protocol

The analysed lecture series is part of the obligatory curriculum of emergency medicine for undergraduate medical students at Frankfurt Medical School. The emergency medicine curriculum consists of a longitudinally structured program with educational units in nearly all semesters of the four years of clinical studies in the six-year program, a structure that is designed to regularly reinforce and increase the depth of understanding of the basic theoretical and practical skills during clinical training [17,18].

The interdisciplinary lecture series is scheduled for 3^rd^ year undergraduate medical students, taking place once per year over an 8-week period from January to March. During this period, the lectures are scheduled twice per week. The lectures cover the main cardinal symptoms of in-hospital as well as out-of-hospital emergency medicine with its algorithm-based treatment and management. Furthermore, topics such as team work and the management of human resources and medical errors are integrated. Depending on the extent of the topic, a single lecture lasts 45 minutes (n = 10) or 90 minutes (n = 11). Four of the 90-minute lectures are conducted by two lecturers together in an interdisciplinary approach. Resulting in a total of 21 lectures.

The students’ attendance of the lectures is optional. However, the lecture series ends with an obligatory 20-item multiple choice examination. Passing the examination is a prerequisite for participating in additional emergency medicine curriculum.

### Measurement

The study measurements took place from January to March 2011 (lecture series 1) and January to March 2012 (lecture series 2). Two months before the second lecture series, all of the participating lecturers received standardised written peer feedback on their lecturing performance. For the peer feedback, two cameras videotaped each lecture. A fixed camera in the back of the lecture hall captured both the slides and the lecturer in the auditorium. The second camera focused directly on the lecturer to capture gestures and facial expressions. The lecturer’s talk was recorded via a microphone tethered to the lecture hall camera.

Each lecture was transcribed into a timeline covering the timing of the different section of each lecture, e.g. introduction and presentation of learning objectives, as well as the existence and duration of interactive parts, e.g. a question and answer section.

In the second step, each lecture was viewed independently by two peer reviewers using a standardized assessment instrument to provide written documentation and feedback. The video reviewer room was equipped with a large TV screen which could display video recordings from both cameras simultaneously on a split screen with optimized tone.

**Figure 1:**
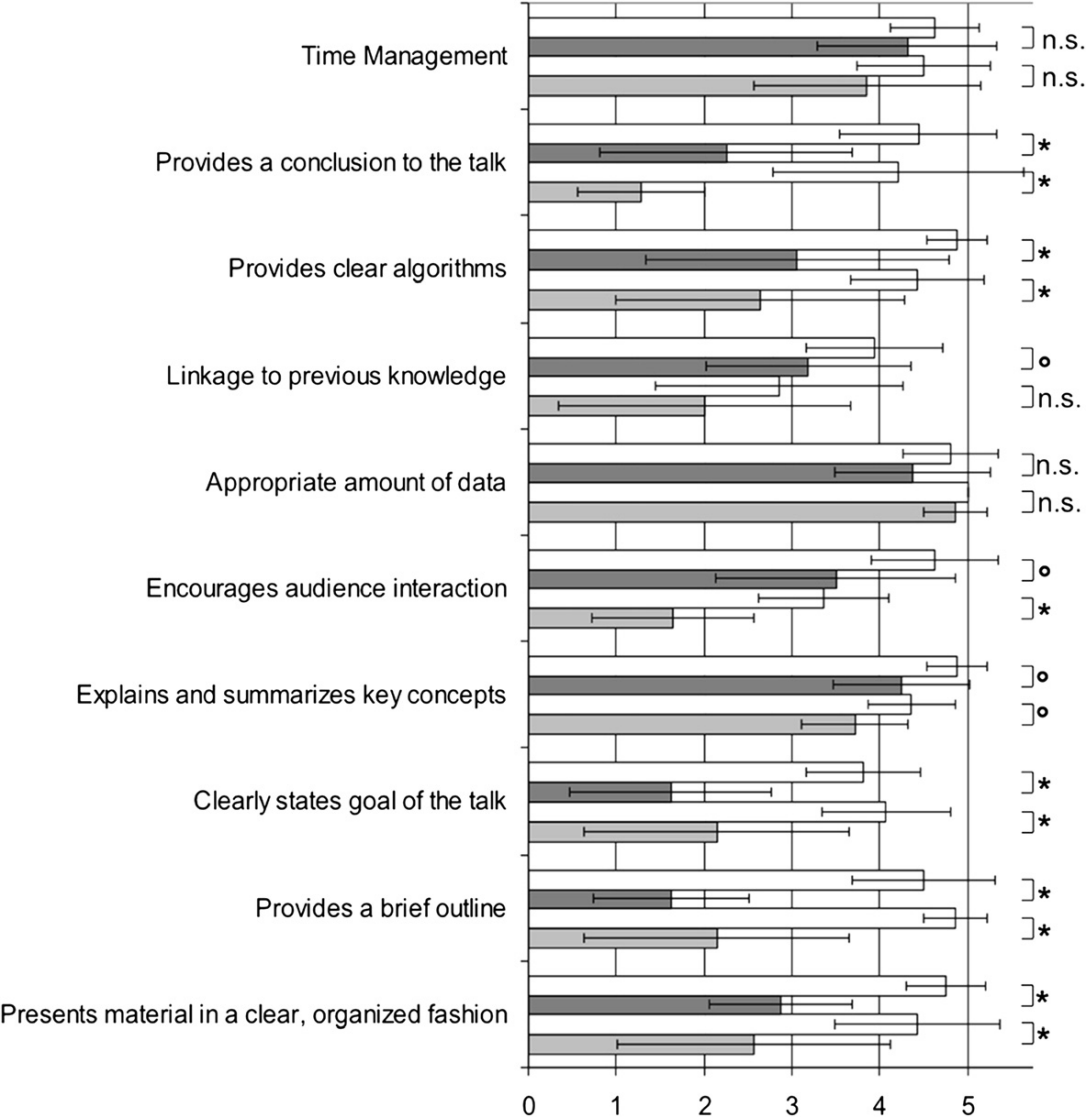
**Ratings for each item in the category ‘Content & Organisation’.** The ratings are presented as the mean ± standard deviation. For the first lecture series, the ratings of the lecturers without didactic training are shown in light grey, and those of the lecturers with didactic training are shown in dark grey. The corresponding results for the second lecture series are shown directly above in the white boxes. Significance of improvement after intervention: *p < 0.005; °p < 0.05; and n.s. = not significant.

**Figure 2:**
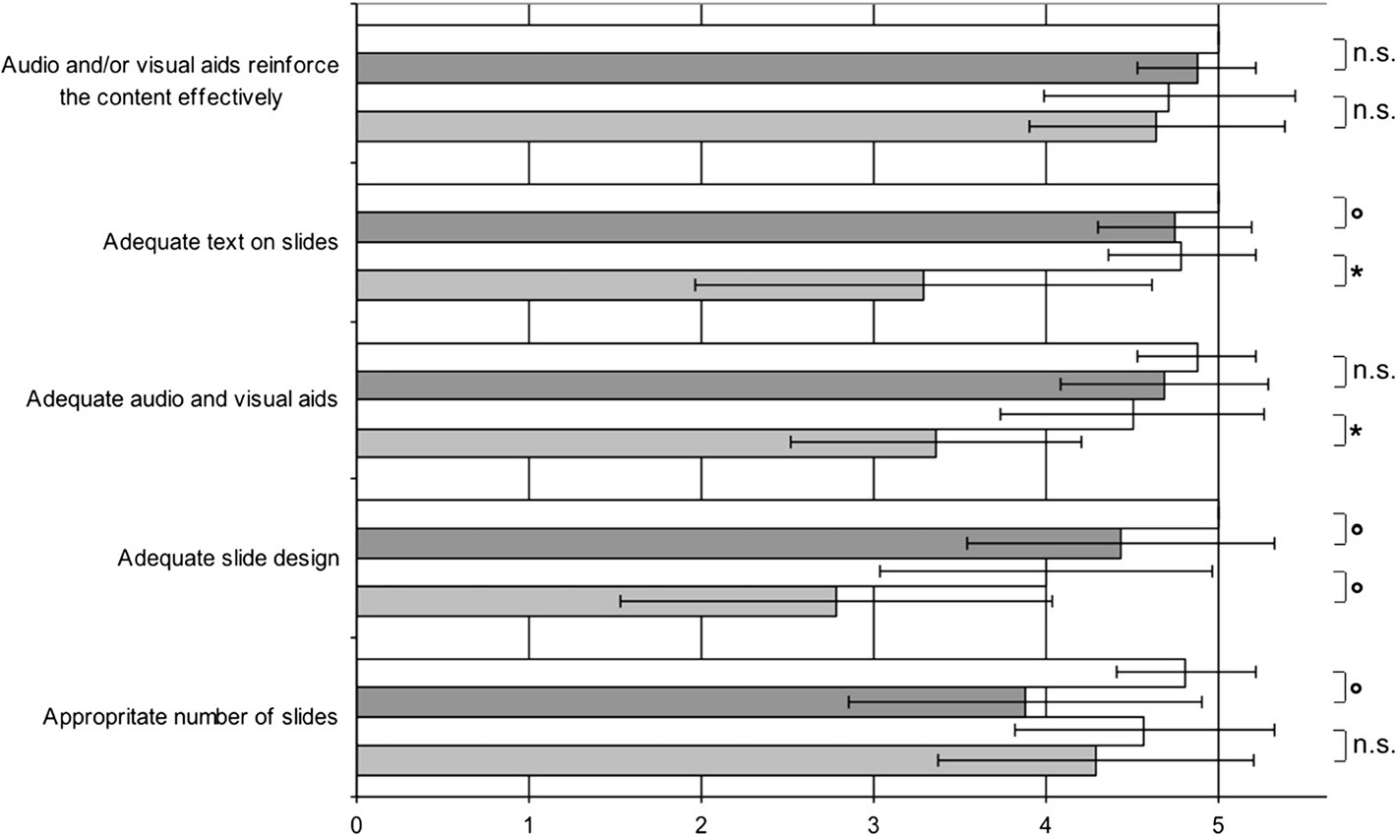
**Ratings for each item in the category ‘Visualisation’.** The ratings are presented as the mean ± standard deviation. For the first lecture series, the ratings of the lecturers without didactic training are shown in light grey, and the ratings of the lecturers with didactic training are shown in dark grey. The corresponding results for the second lecture series are shown directly above in the white boxes. Significance of improvement after intervention: *p < 0.005; °p < 0.05; and n.s. = not significant.

**Figure 3:**
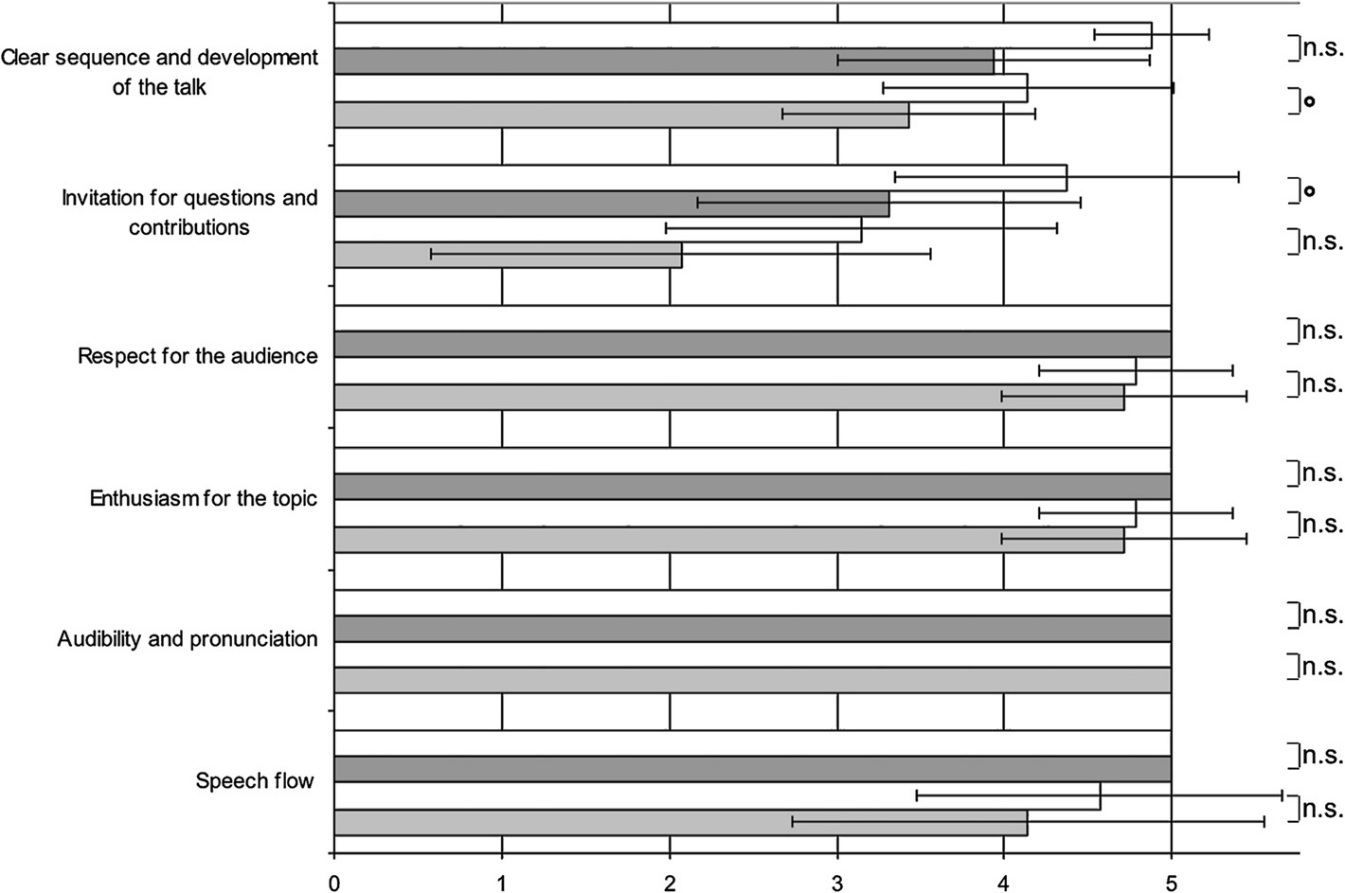
**Ratings for each item in the category ‘Delivery’.** The ratings are presented as the mean ± standard deviation. For the first lecture series, the ratings of the lecturers without didactic training are show in light grey, and those of the lecturers with didactic training are shown in dark grey. The corresponding results for the second lecture series are shown directly above in the white boxes. Significance of improvement after intervention: *p < 0.005; °p < 0.05; and n.s. = not significant.

The assessment instrument was based on the criteria defined in existing literature regarding effective lecturing behaviours, skills, and characteristics [1,6,7,9,12,19-21] and the validated peer assessment instrument for lectures reported by Newman et al. [14,22]. The 21-item instrument is divided into three categories: content/structure (10 items), visualisation (5 items), delivery (6 items) (Figures 1, 2, 3).

Each item was rated on a 5-point Likert Scale (from 5 = excellent demonstration to 1 = does not demonstrate/present/poor) with descriptive benchmarks for the excellent (5), adequate (3) and poor performance (1) rating levels [14,22]. Furthermore, areas of strength were noted, and suggestions for improving weaknesses in lecturing performance were made.

All 4 reviewers were physicians with training in emergency medicine and specific didactic training (postgraduate Master of Medical Education (MME) or currently in a MME program). Herewith, they were acquainted with the assessment instrument because they used the instrument to assess fellow students’ presentations during their postgraduate studies. For this study, all of the raters received an additional 3-hour training session, watching several 15-min examples of previous lectures. They shared their scores and discussed the observed behaviours that had persuaded them to choose a particular performance score for each assessment item. Proper training of the raters is crucial to reduce variability in the instrument’s inter-rater agreement measure by increasing accuracy and consistency of performance assessment ratings [14,22]. During the training, the raters learned to avoid common rater errors (e.g. the halo effect and central tendency) and discussed behaviours indicative of each performance dimension until a consensus was reached [21,23]. Each lecture was reviewed by two raters. The ratings were analysed as described in the ‘data analysis’ section.

The students were regularly asked to evaluate each lecture in emergency medicine with a 3-item questionnaire (overall lecture quality, didactics and delivery/presentation) on a voluntary basis at the end of each lecture using a 5-point Likert scale. These evaluations were used to analyse changes in the lecturers’ evaluations.

In November 2011, two months prior to the beginning of the next lecture series, each lecturer participating in this study received a copy of the lecture observation schedule, the assessment instrument including the written feedback of the raters, and the students’ evaluations.

Each lecture was recorded as described for the first part of the study. The reviewer training, review process and student evaluations were repeated for the second round as described above.

### Data analysis

The statistical analysis was performed using Microsoft Excel for the epidemiological data and evaluation and SPSS 17 for the checklist results. Once Gaussian distribution of the data was verified, the values were presented as the mean ± standard deviation. The Kappa coefficient was computed to determine the inter-rater reliability. The differences in the scores between both groups (no didactic training versus didactic training) were analysed using Student’s *t*-test for independent samples. The differences between the ratings prior to and after the interventions were analysed using Student’s *t*-test for dependent samples.

## Results

Three lecturers declined to participate in the study, and thus these three 45-min lectures were excluded. Two 90-min lectures were excluded due to a defect in the cameras or the recording system. One lecturer left the university hospital after the first lecture series and was replaced. Hence, this lecture was also excluded. Thus, a total of 13 lecturers were assessed (three were assessed twice). Of the 21 lectures in the lecture series, 15 lectures with a total lecture time of 1080 minutes were included and analysed in this study. This includes six 45-min lectures and nine 60-min lectures. The characteristics of the lecturers are shown in Table 1.

**Table 1:** **Characteristics of the observed lecturers**

**Age [years]**	48.4 ± 7.6 (40–65)*
**Sex**	all male
**Status [n]**	
Professor	7
Assistant Professor/PhD	3
Consultant	2
Paramedic	1
**Discipline [n]**	
Anesthesiology	6
Emergency Medicine	1
Forensic Medicine	1
General Surgery	1
Gynecology	1
Internal Medicine	1
Trauma Surgery	2
**Years lecturing for undergraduate students**	
	14 ± 8 (5–33)*
**Years speaking at international conferences**	
	20 ± 7 (13–36)*
**Training/Qualification in Medical Education**	
None	5
Basic educational training°	6
Emergency course trainer^	6
Master of Medical Education	1

*Presented as Mean ± Std.Dev. (Min-Max).

°e.g. One day course ‘Basic university didactics’ offered by the university.

^e.g. Trainer for AHA-, ERC-, ATLS-courses.

Table 2 and Figures 1, 2, 3 show the results of the ratings for the three categories and the respective items for the two lecture series. Except for the category ‘delivery’, there was a significant improvement in the mean score after the intervention in all of the categories (p = 0.002–0.039). When the mean scores of the two groups of lecturers (no didactic training versus those with didactic training) were compared, no significant differences were observed except for the category ‘visualisation’. Here, the lecturers with didactic training received a significantly higher mean score than the lecturers with no didactic training both before and after the intervention (p < 0.05).

**Table 2:** **Peer reviewer ratings of the lectures before (series 1) and after (series 2) written feedback**

		**Lecture series 1**		**Lecture series 2**	
		**No didactic training**	**Training**	**No didactic training**	**Training**
**Overall**	R1	3.29 ± 1.53	3.85 ± 1.39	4.32 ± 0.93*	4.72 ± 0.53*
	R2	3.29 ± 1.53	3.84 ± 1.39	4.29 ± 0.93*	4.71 ± 0.53*
	IRR	0.84		0.84	
**Content & organisation**	R1	2.73 ± 1.56	3.11 ± 1.49	4.16 ± 0.95°	4.52 ± 0.63°
	R2	2.71 ± 1.56	3.10 ± 1.49	4.18 ± 0.95°	4.52 ± 0.63°
	IRR	0.82		0.83	
**Visualisation**	R1	3.66 ± 1.16	4.52 ± 0.56	4.51 ± 0.65*	4.93 ± 0.13*
	R2	3.63 ± 1.16	4.52 ± 0.56	4.43 ± 0.65*	4.95 ± 0.13*
	IRR	0.80		0.80	
**Delivery**	R1	3.96 ± 1.26	4.52 ± 0.89	4.35 ± 0.91^§^	4.87 ± 0.31^§^
	R2	3.96 ± 1.26	4.51 ± 0.89	4.36 ± 0.91^§^	4.83 ± 0.31^§^
	IRR	0.9		0.89	

Data are presented as the mean + standard deviation for each category. Each item within a category was rated on a 5-point Likert scale (5 = excellent demonstration of skill, 3 = adequate and 1 = does not demonstrate).

Significance of improvement after intervention: *p<0.05; °p<0.005, ^§^not significant.

R1: Rater 1; R2: Rater 2; IRR: Inter-rater reliability measured as the kappa coefficient.

In the first lecture series, the lecturers with no didactic training received the worst ratings for the items ‘provides a conclusion for the talk’, ‘encourages audience interaction’ and ‘linkage to previous knowledge’; the lecturers with didactic training received the worst ratings in the categories ‘provides a brief outline’, ‘clearly states goal of the talk’ and ‘provides a conclusion for the talk’. All of these items are included in the category ‘content and organisation’. The items that received the highest ratings and the most significant improvements after the intervention for both groups, ‘provides a brief outline’, ‘provides a conclusion for the talk’ and ‘clearly states goal of the talk’, were also in this category (p < 0.001).

The inter-rater reliability for the pair of raters who observed the same lecture was assessed using the Kappa coefficient. The raters were in good agreement for all of the criteria (0.82-0.9).

Regarding the students’ evaluations, the mean number of evaluation forms completed per lecture in the first lecture series was 59 (range, 47–73), corresponding to a return rate of 22.4%. In the second lecture series, the mean number of evaluation forms completed for each lecture was 51 (range, 41–68; return rate: 18.9%). Table 3 presents the results of the students’ evaluations. The mean student scores for didactics and delivery/presentation were significantly higher for lecture series 2 (after written feedback) than lecture series 1 (before written feedback). This difference was detected despite the fact that different students rated lecture series 1 compared with series2 and the students were blinded towards the presence of the study. The lecturers with didactic training received better ratings than those without training.

**Table 3:** **Student evaluation of the lectures before (series 1) and after (series2) written feedback)**

	**Lecture series 1**		**Lecture series 2**	
	**No didactic training**	**With training**	**No didactic training**	**With training**
**Overall lecture quality**	4.10 ± 0.26	4.43 ± 0.21	4.21 ± 0.43^§^	4.49 ± 0.23^§^
**Didactics**	4.05 ± 0.34	4.38 ± 0.21	4.28 ± 0.43*	4.49 ± 0.27°
**Delivery/Presentation**	4.06 ± 0.33	4.40 ± 0.21	4.23 ± 0.56°	4.55 ± 0.21*

Data are presented as the mean + standard deviation.

Significance of improvement after intervention: *p<0.005; °p<0.05, ^§^not significant.

## Discussion

Lecturing has been criticised as ineffective compared with other methods of teaching that involve students as active participants in the learning process rather than passive observers. This is unfortunate because lecturing is often indispensable, especially for large classes with hundreds of students. Furthermore, when done effectively, lecturing can transmit new information in an efficient manner, explain or clarify difficult concepts, organize ideas and thoughts, challenge beliefs, model problem-solving, and foster enthusiasm and a motivation for learning [2,3]. Didactic lecture will continue to be a mainstay in all parts of medical training [1]. As such, it is important to maintain and improve the quality of lectures.

To our knowledge, this is the first study analyzing the effect of a standardized written feedback on the performance of lecturers in a lecture series. Our results demonstrate that even with a ‘simple’ written feedback, lecturers effectively integrate their newly gained knowledge in future lectures, improving their teaching. Although we hypothesized that positive changes in the lecturers’ performances would occur after written peer feedback, we were extremely surprised by the extent of the improvement since only written feedback was provided, and no additional training occurred. The improvements made were independent of the lecturers’ experience as medical teachers and their prior didactic training.

Our findings are consistent with the existing literature. Providing feedback to faculty members has been shown to clarify performance quality and provide a formative assessment [11,24,25]. It facilitates self-reflection of teaching practices and encourages faculty to discuss their teaching skills and effective instruction [13,25]. In our study, we were able to affirm the existing literature, demonstrating that feedback can help to close the gap between current performance levels and the desired goals of curriculum designers [24,25]. The responses and reactions from the lecturers regarding the feedback were very positive with several lecturers completely revising their lecture (e.g. new slides, figures, and/or videos). These findings are consistent with the recent qualitative study of McLeod et al., demonstrating that all participants receiving peer review enthusiastically endorsed the benefits of peer assessment [16].

However, effective peer assessment of teaching should be criteria-based and use instruments that produce highly reliable measures [9,14]. For this reason, in addition to a validated assessment instrument, adequate rater training is essential to ensure that all of the raters have internalized the rating standards and are committed to giving the necessary time and effort [14,22]. In addition to using the assessment instrument during their postgraduate training, the raters discussed the rating standards during the rater training, using videos of former lectures. They focused on those items identified as difficult in both the literature and training to reach a consensus. The clear, descriptive benchmarks for the excellent, adequate and poor performance rating levels provided by Newman et al. [14,22] helped in this area, facilitating powerful feedback by the raters. Thus, the raters were in agreement on all of the criteria in our study.

During the first peer assessment, the main deficits were in the items of the category ‘content & organisation’. After written feedback, this category showed the greatest improvement, with highly significant improvement in 6 of the 10 items. The items ‘provides a brief outline’ and ‘provides a conclusion for the talk’ showed the greatest improvement.

The smallest improvements were found in the category ‘delivery’. This category includes items such as ‘speech flow’ and ‘enthusiasm for the topic’. These items are innate to an individual and cannot be changed as easily without additional training compared with items in the other categories. We hypothesize that practical training e.g. with video feedback and clear information about the expected behaviour would have a higher impact on these items than written feedback. With respect to the raters, we were able to demonstrate that an agreement on a definition of ‘expected behaviour’ for these items can be achieved. To gain deeper insight into this specific area, further studies are needed.

Although the students were blinded towards the study, they noticed and appreciated the efforts of those lecturers who changed their lecture habits; several added under the “comments” section of their teacher evaluation that they appreciated the inclusion of clear learning objectives, an outline of the purpose and contents of the lectures and the manner in which questions were encouraged and addressed.

Both the students in lecture series 1 and lecture series 2 rated those lecturers with didactical training higher than those without training despite being blinded to the training experience of their lecturer. Thus, students are able to differentiate between lecturers with different teaching abilities.

We have regularly asked all students to evaluate each lecture and have provided the detailed results to all lecturers since 2005. However, we found only small improvements in the quality of the lectures following the students’ feedback. These findings are consistent with the statement of Newman et al. [14] arguing that the faculty undergoing a review needs to trust that the ratings are not idiosyncratic scores but reflect their actual performance. This means that the assessment instrument must be reliable as measured by inter-rater agreement, the rater must be respected and the feedback must be as specific as possible in its items and provide points of action [14].

This study has some limitations because it was conducted at a single medical school with only one study sample of lecturers in emergency medicine, which might restrict its explanatory power and its transferability to other medical schools. However, this limitation does not diminish the significance of the results and the pronounced impact of the written peer feedback on the performance of most of the lecturers. It may serve as a model for the development of similar programs in all levels of medical training to improve instructional effectiveness.

The fact that the reviewers rated a lecturer based on a videotape of the lecture rather than a live presentation is also a limitation. Providing feedback based on videotaped teaching sessions can be criticised because the real environment and atmosphere cannot be completely captured [22]. Furthermore, it cannot be guaranteed that the raters are not disturbed (e.g. answering a phone call) or watch the video in discontinuous segments. However, our results regarding inter-rater reliability have acceptable high levels for all of the items.

This study does not investigate the lecturers’ self-assessments and compare self-assessments to peer ratings. To gain an insight into this area and the effect of peer feedback on self-assessment, future research is needed.

## Conclusion

This study demonstrates the significant impact of a single standardized written peer feedback on lecture quality. Based on this study, the assessment instrument and study design will be used as a basis to evaluate and improve additional lecture series in other disciplines at our medical school.
